# Supplementary material for: Emergency department is not safe anymore: Nurses describing their suffering
Source: PLoS One. 2025 Apr 29;20(4):e0322704. doi: 10.1371/journal.pone.0322704 (PMC12040212; doi:10.1371/journal.pone.0322704)
Supplement: S2 File — (DOCX) [file pone.0322704.s002.docx]

**Full interview script**

**Case #7**

| رقم السؤال | **صيغة السؤال** | **الجواب** |
| --- | --- | --- |
| 1. | عمرك تعرضت للعنف اثناء عملك في قسم الطوارىء؟ | نعم لفظي كثير و جسدي و انا اتعرضت للعنف مره واحده جسديا |
| 2. | احكيلي عن هذه التجربة بتفاصيلها | الاكشن كان انه بتعرفي انت الناس تفكر اي حدا لابس كوت هو دكتور بيؤدي نفس الشغل, كان مرافق المريض يستنى الدكتور على اساس انه هو حاط بباله انه الدكتور متاخر عليه ويومها الام او المريضة صار معها زي  sometime female .especially old age ببالغو شوي seeking attention, ودغري رمت حالها على الارض و يومها ركض ما بده نشيلها عن الارض وهو اول واحد اجى في باله يفش غله فينا, بتعرفي انه احنا الواجهه الاولى او بوز المدفع , مش انه ضربني لا حط ايده على صدري و دزني وتلاسنت انا واياه واخذت الاكشن الصح قدمت فيه شكوى , بالمستشفى و هرب من المستشفى لكن انجاب وصلنا لي رقم تليفونه خلال ربع ساعه ثلث ساعه كان عندنا في قسم الحوادث قدمت شكوى رسميه صار يطلع مبررات ويعتذر وانه امه تعبانه ،انا اشفقت عليه كرامة للحجه الي معه هو فعليا ما سبب لي هذاك الاذى بس انه حط ايده على صدري واذني يا دوب سنتيمترات بسيطه انا كنت واقفه قباله كنت جاي بدي اشيل الحجه اجي وهجم علي انا وقتها ما التفت له كان همي اشيل الحجه عن الارض يعني صيحت عليه لكن هو رجع هجم على الحجه ما حدا يصيبها ما حدا يقرب عليها يعني حتى مع الضربه كملت شغل مع الحجه واجينا نرفعها. |
| 3. | ما هو شعورك عند التعرض للعنف في قسم الطوارىء | انا تضايقت كثير هديك اللحظه لاني مش مقصر بشغلي , ما بطلعلك تمد ايدك وهذا الشيء كله انكتب في محضر رسمي عند مندوب الحوادث وحكاله اذا اعتذرت من الممرض ممكن تنحل ممكن تدخلو نحكي معه حتى تسحب الشكوى اما اذا ما اجيت اعتذرت راح يصير بحقك شكوى رسميه ومحاكم وهو لوما احس بالخوف و راجع نفسه واستخطاها هو كان كثير معصب وكان السبب هو جهل الناس ونظرته انه هذول المداومين في المستشفى كلهم واحد وهو في كمان ثقافه عند الناس سائده انه اطلع على الكوادر الطبيه بالصوت العالي و خذوهم بالصوت, بعدين خلصنا كرامته للحجه , هو الواحد اكيد عصب حسيت حالي sweaty شوي تنشن تكي كارديا, كثير ضليت احافظ على هدوءي لكن داخليا مرتبك قاعد بغلي من جوا الشغله هي عصبيه وnervousness اكثر من ما هي depression او sadness, يعني عدم المؤاخذه ما فينا واحد عاجز كل واحد بيقدر ياخذ حقه بيده لكن الواحد حابب يمشي بالقانون legally. |
| 4. | كيف كنت تتكيف مع واقعة العنف عند حدوثها | بنفس اللحظه امتصيته و سكتت نحن عندنا في المستشفى في الوايت كود هذا الكود بتفاعل لما يصير فيه مشكله او actual or potential violence,مثلا واحد حس انه بده يصير ضرب او تكسير بنطلب الكود او واحد بلش يصيح نطلب الكود .وبيجي عليه امن المستشفى واذا كان الاشي كثير كبير بيجي مندوب الحوادث . |
| 5. | عرف العنف ضد الممرض في الطوارىء | العنف بالذات عندنا هون في الاردن و قسم الطوارئ ما بتقدري تصوري بتعريف لانه عندك عندك تعدد في الثقافات different culture , different believes ,different types of perceptions,عند الناس عشان هيك ما بتقدري تحصر العنصر مرات العنف اللي بيصير على الكادر بسبب تقصير الكادر نفسه مش دايما الموقف situation بكون نفس event نفس السبب ونفس الدافع الى خلي الشخص يبدر منه العنف لكن بشكل عام ممكن نحكي هو استخدام القوه استخدام لفظي استخدام الشتائم حتى انه الواحد يحصل على الخدمه اللي حاطها في باله. |
| 6. | عرف كل نوع من انواع العنف | اللفظي هسه اوقات في ناس مثلا بيجي بده خدمه معينه منك وخاصة في قسم الطوارئ مثلا وهو يعاني من مشكله من سنوات انت بتحكي له لازم يراجع العياده او مثلا بده خدمه ما بتتسوي في الطوارئ مثلا بده عياده الجلديه يعني عياده الجلديه يعني اي مشكله جلديه بتكون من سنوات مش حاله طارئه المفروض انك تراجع فيها العيادات في ناس عندك ما بدها تستنى في عندنا في المستشفى قسم ترياج في لو نظام و سستم هذا بطبق تصنيف المرضى حسب الاولويه حسب severity or priority,اغلب شعبنا ما بدو يستنى يعني بكون عنده simple case يعني شيء بسيط و بده يمشي قبل اكثر واحد تعبان بالطوارئ واجبك ان كانت تعمل تصنيف للمرضى حتى لو حكي و صيح لازم ماتاثر عليك لكن في ناس بده يصيح هسه بدك تمشينا خاوه هاي وحده من انواع العنف او مثلا مريض بستنى باجراء طبي مثلا سحب عينات مختبر بده العينه تطلع في خلال خمس دقائق هي مثلا بدها تقريبا نصف ساعه بهاي الفتره هو بيعتبر انه التقصير من كف بيجي بوجهك حكي والصياح ومرات مسبات نحن هون بدورنا لفظيا مدافع عن حالنا يعني مش كل مره بنتصل على الكوود بدك تعرف كيف تحتوي الموقف وتكون لك شخصيتك دافع عن حالك و توقفه عند حده انه لما يجي المره الجاي ما يستخدم نفس الاسلوب هذا معنا.  -وفى نوع هو sexual او الجنسي :some times ,بيصير بصراحه احنا عندنا هون في القسم مراجع male يتحرش في female نادر اللي بيصير اللي هو العكس female تتحرش في male,على الصعيد الشخصي صارت معي اكثر من مره ممكن حد ثاني ما يستوعب اللي صار بس اكثر من مره بتكون عارف انه في شيء غلط بس انت بتعمل cutting الموضوع وقطع . |
| 7. | اكثر نوع يتعرض له الممرض في قسم الطوارىء | هو اللفظي اكثر بنتعرضله |
| 8. |  |  |
| 8-1 | اسباب تتعلق في بيئة العمل ما | هسه اللود في المستشفيات بشكل عام .احنا كونا  Thirtiary health center, مستشفى كبير مش دايما المرافق متوفره للناس كلها الناس بتيجي على قسم الطوارئ بكون فيه 20 مريض بالقسم هوبيفترض انه هذول العشرين جايين نزهه مش مرضي بس هو المريض في ناس قبلوا و دورهم قبله في ناس priority اكثر منه ويشرح له انه هذول لهم اولويه اكثر منك لكن هو ما بده يستوعب لكن هو معتقداته للاسف في المعتقدات الخاطئه او bad culture or manner السيئه خلينا نحكي, فهو بيجي بده يمشي خذوهم بالصوت بلكي خافوا هاي وحده من الاشياء شغله ثانيه مثلا تعرفي في قسم الطوارئ بنبعث فحوصات بنودي مرضي بتصور و صور اشعه في صور تظهر على النظام لكن بدها report مثلا U.S or C.T,هاي بتاخد وقت اكثر من قيم الاشعه في صوره والاخصائي قيم الصوره ويكتب التقرير فهو الا بده هسه ما بده انتظار ويتنج بالنسبه للازدحام حتى لو صار ازدحام نحن عندنا القدره انه مانخلي ازدحام يعني شو ماكان شفت انتم الطوارئ شو ماكان وضعك و بتعرفوا انتم تمشو اموركم لانه نحنا مش قصدا بدنا ناخر حدا, نحن بنشتغل اول باول اوردرات ما بناخر ولا بنراكم , لانه بالنهايه هذا الشغل علينا علينا في الشغل انت مشتغله بتشتغلوا بكير احسن . |
| 8-2 | اسباب تتعلق بالمريض والمرافقين | هو مش ممكن هو 99 بالميه هو سبب العنف في مثل بيقول صاحب الحاجه ارعن بمعنى اخر هو جاي بفكر انه لازم 30 ممرض 30 دكتور لازم يكونوا عند امه او ابوه وما يفارقوا يظلوا عنده ،فانت خلص بتشتغلي الشغل اللي عليك وبترجع عالكونتر حتى تقدم خدمه لمريض ثاني فهو بيجي بده اياك انت تضل عنده، هو خلص مشكله mistrust ,اللي بتصير بين المرافقين وبين الكوادر الطبيه خطا طبي تقصير معين هذا للاسف بتعم مع الكوادر جميعها يعني مشكله صارت بالكرك نحن شو دخلنا هون يتعمم علينا هو يحط في باله انه خلصهم كلهم في كل مكان مقصرين شو دخلنا فاذا تاخرت عليه نص دقيقه راح يفجر فيك هو جاي وحاط في باله انك انت انسان مقصر ما بدي اقوم بواجبك زي ما قلت لك هو جاي محضر حاله في يعني في ناس بيجي بوقف على الطاوله بصير اصيح, يعني انا قبل فتره اجي مريض عينه بتوجعه بقول له سلامتك صحيح علي بقول لي لا تقول لي سلامتك جيبلي الدكاتره جيبلي الدنيا شو سلامتك عيني بتوجعني من ثلاثه ايام جاي الساعه ثلاثه اربعه الفجر ما في نفس اللحظه خلاص هاي اللي زي هيك ما بنفع تقول له هدي حالك اذا سمحت بدك تيجي لكل لكل فعل رد فعل، بصراحه انا قلت له انك ما كمل 30 ثانيه وانت واقف صرت تصيح ما حد اتاخر عليك ،فانا لما رديت عليه بنفس الطريقه سكت ما سمعنا صوته وسكت، هو لازم انك تحتوي الموقف في البدايه لكن مش دائما بتزبط انك تحتوي الموقف خصوصا اذا واحد جاي ومستشر مش محترم حدا .. |
| 8-3 | اسباب تتعلق بالممرض | هاي مرات ممكن يصير تقصير من شدة load على الممرض الممرض يتاخر شوي في تقديم الواجب .هذيك الساعه ممكن تتاخر على المريض مش قصدك انك تتاخر او تقصر مرات بيجيك 45 اوردر وكلهم بدك تسويهم مع بعض هاي اكيد ما راح تشتغل هم كلهم مره واحده اكيد راح تشتغل واحد واحد ممكن اخر واحد تيجي عليه ممكن تكون متاخر عليه طبعا  -طبعا نقص الكادر بشكل عام يؤدي الى العنف worldwide problem,ممكن نحكي انه بسبب الثاني او الثالث من اسباب العنف |
| 9-1 | ما هي عواقب العنف على الممرض نفسيا و جسديا | ممكن ان يتعرض لاذى جسدي اكثر من الاذى النفسي ومع التراكمات اكيد في ناس بتصير تكره المهنه بفقد ثقته بالمهنه في ناس بطلت تحبها في ناس .بتحسي الممرض او الدكتور بفقد dedication المهنه في ناس صار معها مواقف غيرت تخصصاتها ومسار عملها واستقالت |
| 9-2 | ما هي عواقب العنف على المريض | دائما بلاحظ شغله انه الانسان اللي بيجي بضرب موظف وبصيح على موظف او او بفتعل المشاكل بتحسي استغفر الله العظيم ربنا من فوق بعطل شغله وعيناته تتاخر او ما بتزبط او بدهم يعيدوها .  شغله ثانيه انك ممكن ما تقدم له العنايه الطبيه بنفس الجوده لحد ثاني هسه جوده care والعنايه المفروض انها ما تتغير بس لما يكون مرافقا ومريض كثير وقح لدرجه انه في مرات بنات بخاف فوتوا يقدموا الخدمه او تنفذ اوردر ممكن تنتظر ممرض شاب معها يكون يعمل شغله ثانيه حتى يستنى شغلته ويقدم الخدمه بدلا منها ممكن يتاخر على المريض وفعليا ممكن البنت تتعرض للضرب لكن الشاب لو راح ممكن انه يخافوا منه ممكن يغير رايه |
| 9-3 | ما هي عواقب العنف على بيئة العمل | اكيد يعني صارت مشكله صار الصياح المريض الي بحدد وراح يتاثر بصير فوضى بشكل عام في القسم الناس تتجمع تعرفي احنا عندنا ناس كتير curious في حال سمع صوت الصياح او اي شيء الكل بهجم الكل بصير يتفرج بس شوف ايش في بصير نوع من السترس او الضغط يؤثر على الممرض, بضل افكر بنفس الموقف اللي صار معه وطبعا بصير بيئه غير امنه تيجي على الشغل في لحظه تكون مداوم وصار معك عنف بتحس بخلص على نفسك يعني في مشكله صارت في المستشفى قبل ثلاث سنين, طخوا على الامن بدون ذكر المنطقه والاسماء دخلوا على المريض الغريم تبعهم على العمليات فاتوا عليه في هذا الوقت صارت انه بيئه غير امنة او un safe environment or hazardous |
| 10 | ما هو التدريب والمعلومات المقدمة للمرضين لمحاربة العنف | انا برايي لازم الدورات او التوعيه تنعمل لالعائله او المرافقين مش للتمريض يعني نحن في السلامه العامه هون بيعطوا دورات منشورات توعويه في سياسات داخليه بتقلل من العنف, شو لازم تعمل وكيف بتخبر مسؤول , كيف تخبر in charge نيرس وكيف تكتب تقرير incident report حدا بنضرب بده المستشفى يكون معه خبر حتى تحمي حالك . |
| 11 | ما هو دورك عند حصول العنف ضدك او ضد اي ممرض في الطوارىء؟ | بتدخل وبحاول احل المشكلة |
| 12 | ما هي الاجراءات المتبعة في المستشفى للتعامل مع حالات العنف ضد الممرض؟ | دور المستشفى اذا صار في شكاوى هذا اكيد محامي المستشفى على درايه ورئيس الدائره اللي صار فيها المشكله مدير المستشفى لازم يتبلغ حتى يقدر يدافع عن الموظف اذا مظلوم او ما كان على حق |
| 13-1 | اجراءات في بيئة العمل | في الوايت كود هسه نحن عندنا كاونتر الجراحه وكان ترياج كله ازاز يعني اذا حدا بده يضرب او حدا او حدا بده يرمي شيء يمكن نسبيان هاي تحمي شوي الممرض ممكن يخلوا مثل الكاونترات يكون مغلق تماما بحيث انه الممرضين يعلق على حاله لو المرافق بده يضربه ما بقدر يوصلوا |
| 13-2 | اجراءات من قبل المستشفى والقوانين المتبعة | اكيد طبعا اذا كان العنف سبب بطئ في الاجراءات او انه المرضى ما بدو يستنى فعليا ممكن ايجاد اليات او حلول جديده حتى تخفف من الضغط او الانتظار للمريض |
| 13-3 | اجراءلت لتوعية المجتمع | هسه المستشفي في له صفحه على الفيسبوك يفترض يصير ينزل منشورات توعويه عن الصحه يعملوا دورات للناس وزعوا منشورات توعويه على الصفحه مثلا حدا هيك من الناس اللي عمل مشكله يتخذ اجراء صارم ممكن يكون اجراء رادع للناس اللي حواليه لانه الحكي متداول بين الناس |
| 13-4 | اجراءات للممرض | ممكن اذا في نقص في الكوادر يفترض انه يزيد عدد الكوادر حتى نحد من مشكله التاخير للمريض |
