# Supplementary material for: Emergency department is not safe anymore: Nurses describing their suffering
Source: PLoS One. 2025 Apr 29;20(4):e0322704. doi: 10.1371/journal.pone.0322704 (PMC12040212; doi:10.1371/journal.pone.0322704)
Supplement: S3 File — (DOCX) [file pone.0322704.s003.docx]

|  |  | **Full interview script**  **Case #7** |
| --- | --- | --- |
| 1. | Were you exposed to violence while working in the emergency department? | Yes, a lot of verbal and physical abuse, and I was subjected to physical violence once. |
| 2. | Tell me about this experience in detail | The action was that you know, people think that anyone wearing a coat is a doctor doing the same job. The patient’s companion was waiting for the doctor, thinking that he was late, and that day the mother or patient had the same thing happen to her.  And immediately she threw herself on the ground and that day he ran, he didn’t want us to lift her off the ground and he was the first one who came to his mind to vent his anger on us, you know that we are the first front or the point of the cannon, not that he hit me but he put his hand on my chest and pushed me and we fought and I took the right action and filed a complaint against him at the hospital and he ran away from the hospital but Injab reached me his phone number within a quarter or a third of an hour he was with us in the accident department, I filed an official complaint and he started making excuses and apologizing and saying that his mother is tired  I felt sorry for him out of respect for the argument he had with him. He actually didn't cause me that harm, but he put his hand on my chest and my ear, which was only a few centimeters away. I was standing in front of him. I was going to pick up the argument, but he attacked me. At the time, I didn't pay attention to him. My concern was to pick the argument up from the ground. I shouted at him, but he came back and attacked the argument. No one should hit it, no one should get close to it. I mean, even with the blow, I continued working with the argument and we came to pick it up. |
| 3. | How do you feel when you are exposed to violence in the emergency department? | I was very upset at that moment because I was not neglecting my work, you did not raise your hand and all this was written in an official report with the accident representative and he told him that if you apologize to the nurse it might be resolved you can go in to talk to him until you withdraw the complaint but if you do not come and apologize there will be an official complaint and courts against you and if he did not feel afraid and reconsidered himself and overlooked it he was very angry and the reason was the ignorance of the people and his view that these people on duty in the hospital are all the same and there is also a prevailing culture among people to loudly address the medical staff and take them by voice, then we finished his dignity for the argument, he definitely got angry I felt a bit sweaty and tense, I kept maintaining my calm a lot but inside I was confused and seething inside the matter it is nervousness and nervousness more than depression or sadness, I mean no offense none of us is helpless everyone can take his right In his hand, but one wants to walk legally. |
| 4. | How did you cope with the violence when it happened? | At the same moment, I absorbed it and kept quiet. We have a white code in the hospital. This code reacts when there is a problem or actual or potential violence. For example, someone feels that there is going to be a beating or smashing, we ask for the code, or someone starts crying, we ask for the code. And the hospital security comes to him, and if the matter is very serious, an accident representative comes. |
| 5. | Define violence against nurses in emergency situations | Violence, especially here in Jordan and the emergency department, you cannot imagine a definition because you have a variety of different cultures, different believes, different types of perceptions among people. That is why you cannot limit the element. Sometimes the violence that occurs to the staff is due to the negligence of the staff themselves. It is not always the situation. It is the same event, the same reason, and the same motive that made the person emanate violence. But in general, we can say that it is the use of force, verbal use, the use of insults, so that the person gets the service he had in mind |
| 6. | Define type of workplace violence | Verbally, sometimes people come to you, for example, wanting a specific service, especially in the emergency department, for example, and he has been suffering from a problem for years, and you tell him he needs to visit the clinic, or for example, he needs a service that is not provided in the emergency room, for example, he needs a dermatology clinic, meaning a dermatology clinic means any skin problem that has been around for years is not an emergency case, you are supposed to visit the clinics there, there are people who don’t want to wait, in our hospital, there is a treatment department, in which there is a system that applies the classification of patients according to severity or priority, most of our people don’t want to wait, meaning they have a simple case, meaning something simple, and they want to leave before the most tired person in the emergency room, your duty, if you are categorizing patients, even if they talk and cry, it must not affect you, but there are people who want to cry now, you need to leave us, brother, this is one of the types of violence, or for example, a patient is waiting for a medical procedure, for example, taking samples from a lab, he wants the sample to come out within five minutes, for example, it takes about half an hour During this period, he considers negligence as a slap, then he comes in your face, talking, shouting, and sometimes cursing. Here, we, in turn, verbally defend ourselves. I mean, not every time we call the code, you need to know how to contain the situation and have your own personality. Defend yourself and stop him so that the next time he comes, he doesn't use the same method with us. -And there is a type that is sexual: sometimes, honestly, it happens. Here in the department, a male client harasses a female. It rarely happens. The opposite is true for a female client harasses a male. On a personal level, it happened to me more than once. It is possible that someone else wouldn't understand what happened, but most of the time, you know that something is wrong, but you keep blocking the issue. |
|  | The predisposing factors of WPV/ Reasons related to the work environment | Now, the load in hospitals in general. We were a Thirtiary health center, a large hospital, the facilities are not always available for all the people. People come to the emergency department, and there are 20 patients in the department. He assumes that these 20 are coming for a walk, not just patients. He is the patient. There are people who have been accepted and their turn is before him. There are people who have more priority than him. He explains to him that these people have more priority than you, but he does not understand. However, his beliefs, unfortunately, are wrong beliefs or bad culture or bad manners. Let's talk. So he comes and wants to walk. Take them by voice, maybe they are afraid. This is one of the things. It is another matter. For example, you know that in the emergency department, we send tests. We bring patients in for imaging and x-rays. These images appear on the system, but they need a report, for example, U/S. or C/T. These take more time than the x-ray values ​​in the image, and the specialist evaluates the image and writes the report. He is the one who needs to wait now. Regarding the crowding, even if it happens Congestion We have the ability to avoid congestion, meaning whatever you see, you are the emergency, whatever your situation, and you know how to manage your affairs because we do not intend to delay anyone. We work on orders as soon as possible, we do not delay or see you, because in the end this work is on us. It is on us at work. You are busy, so work early is better. |
|  | The predisposing factors of WPV/ Reasons related to the patient and companions | It is not possible, he is 99% the cause of the violence. As the saying goes, the owner of the thing is reckless. In other words, he came thinking that 30 nurses and 30 doctors must be with his mother or father and must not leave him. You finish doing your job and then go back to the counter to provide service to another patient. He comes and doesn’t want you to stay with him. He finished the problem of mistrust. What happens between the escorts and the medical staff, a medical error, a specific negligence, unfortunately this is common with all the staff. I mean, a problem that happened in Karak. What do we have to do with it? It is generalized to us. He puts in his mind that he finished them all. Everywhere they are negligent. What does it have to do with us? If you are late for him for half a minute, he will explode at you. He came and put in his mind that you are a negligent person who does not want to do your duty, as I told you. He came prepared. I mean, some people come and stand at the table and start crying. I mean, a while ago, I came sick and When he is in pain, I tell him, "Get well soon." He tells me, "Don't tell me, 'Get well soon.' Bring me the doctors. Bring me the world. What is 'Get well soon.' My eye has been hurting for three days. He's been coming at three or four in the morning, not at the same time." Enough with the kind of person like that. You don't tell him, "Calm down, please." You have to come. Every action has a reaction. Honestly, I told him, "You didn't even complete 30 seconds standing. You started crying. No one was late for you." When I responded to him in the same way, he kept quiet. We didn't hear his voice and kept quiet. You have to contain the situation at the beginning, but it's not always possible to contain the situation, especially if someone comes as a consultant and doesn't respect anyone. Sometimes, there might be negligence due to the overload on the nurse.  . |
|  | The predisposing factors of WPV/ Reasons related to the nurses | The nurse might be a little late in delivering the task. At that hour, you might be late for the patient. You don't mean to be late or negligent. Sometimes, 45 orders come to you and you have to do them all at once. This definitely won't work. They will all be done at once. You definitely will work one by one, maybe the last one. You might be late to it, of course.  - Of course, the lack of staff in general leads to violence worldwide. We can say that it is due to the second or third reason for violence |
|  | What are the psychological and physical consequences of violence on the nurse? | He may be exposed to more physical harm than psychological harm, and with the accumulation of things, there are certainly people who begin to hate the profession and lose confidence in it. There are people who stop loving it. There are people who make the nurse or doctor feel that they have lost dedication to the profession. There are people who have gone through situations that made them change their specializations and work path and resign. |
|  | What are the consequences of violence on the patient? | I always notice that when a person comes and hits an employee, shouts at an employee, or causes problems, you feel, God forbid, that God from above is disrupting his work and his samples are delayed or don't work or they want to redo them. Another thing is that you might not provide him with the same quality of medical care until now. The quality of care and attention should not change, but when he is accompanying a patient, he is very rude to the point that sometimes girls are afraid to come in to provide the service or carry out an order. They might wait for a young nurse with her who is doing another job until he waits for his job and provides the service instead of her. He might be late for the patient, and in fact, the girl might be exposed to beating, but if the young man goes, they might be afraid of him and might change his mind |
|  | What are the consequences of violence in the workplace setting? | Of course, a problem has arisen. The patient will scream and be affected. There will be chaos in the department. People will gather. You know, we have a lot of curious people. If they hear screaming or anything, everyone will attack. Everyone will watch. But see what happens. A kind of stress or pressure affects the nurse. I keep thinking about the same situation that happened to him. Of course, it becomes an unsafe environment. You come to work at a moment when you are on duty and violence happens to you. You feel like you are going to finish yourself off. I mean, there was a problem in the hospital three years ago. They attacked security without mentioning the area or names. They entered the patient, their enemy, in the operating room. They invaded him. At this time, it became an unsafe environment. |
