# Supplementary material for: Emergency department is not safe anymore: Nurses describing their suffering
Source: PLoS One. 2025 Apr 29;20(4):e0322704. doi: 10.1371/journal.pone.0322704 (PMC12040212; doi:10.1371/journal.pone.0322704)
Supplement: Attachment — Submitted filename: 17.3.2025.PLOSONE.Rrespon to Reviewer.3.2025 .docx [file pone.0322704.s005.docx]

| Reviewer # | Reviewer comment | Response to reviewer comment |
| --- | --- | --- |
| **Editor** | 1. Please ensure that your manuscript meets PLOS ONE's style requirements, including those for file naming. | PLOSE one guidelines were followed all over the study |
|  | 2. please ensure that you have specified (1) whether consent was informed and (2) what type you obtained (for instance, written or verbal, and if verbal, how it was documented and witnessed). | Each participant were approached and contacted separately and were verbally consented for their participation. Each participant signed a consent form after reading the consent form document. Data collection section  In addition they verbally approved to participate and disclosed their experience of WPV. Each participants signed a a written consent form before the interview. |
|  | 3.  Your ethics statement should only appear in the Methods section of your manuscript. If your ethics statement is written in any section besides the Methods, please delete it from any other section | Ethical statement is in the ethical consideration section. And was deleted from other sections  Before data collection, institutional research board (IRB) approval was acquired from university (x) hospital and Ministry of Health (IRB: 147/132/2020). |
|  | 4.  Please include captions for your Supporting Information files at the end of your manuscript, and update any in-text citations to match accordingly. | - an interview script example was attached to the manuscript file |
|  | 5.  Please review your reference list to ensure that it is complete and correct. | Reference list was revised, and they are complete and correct. Some references are updated |
|  |  |  |
|  | The conclusions must be drawn appropriately based on the data presented. | The conclusion was changed and reflected study findings accordingly. See occlusion section |
| **Reviewer#1** | It would be better that the paragraph 5 (In recent years, WPV increased in Jordanian hospitals ….) to follow the paragraph 3 (Nurses who work in the Emergency Department (ED) are one of the…). | The change was made . see introduction section  Also editorial changes , and introduction flow was changed |
|  | *Results Table 2 needs some modification | Table 2 was update . see table |
| **Reviewer #2:** | -overall it needs extensive English editing | Editing was made all over the manuscript |
|  | refrenced study results are included that must be in the discussion section, line 81 provide like recommendations for planning educational intervention line 87 describes the present study result not an aim for the study | The statements were revised and changed  Please see the introduction |
|  | Methods lacks the limitations for the qualitative approach as researcher bias, also it lacks hospital characteristics that could affect WPV | The limitation were revised and updated and written at limitation section |
|  |  |  |
|  | what is the participants selection method used that gurantee "no bias" | The qualitative study used purposive sample that was used nurses who disclosed the experience of WPV . this approach sure the representativeness of phenomenon . and the aim of the qualitative research is to describe people experience . the generalizability is one of its limitation  Please see limitation section |
| **Reviewer #2:** | The Colaizzi method need to be discussed in more details | The steps are discussed in table 1. See table |
|  | Results: no clear explaination of how themes were derived | See steps of Coliazzi of data analysis and how the PI derived the study themes . table 1 |
|  | Discussion need to be more concise and focused to offer deeper interpretation as it focuses more on repitative comparisons without giving researcher's opinions why similarities or differences exist | Several changes done including emerging some ideas , resynthesizing and relating findings from similar literature .  See discussion section |
|  | Limitations: does not show how these limitations impacted the study | The study limitation was updated and revised  See limitation section |
|  | Conclusion: also too much repititions it need to be more concise | The confusion was changed and updated . see occlusion |
|  | some references are too old | 18 references are within 7 years  Some references are updated you can compare with the previous draft  Also refrence list updated based on PLOSE one guidelines  -Some references are original for example Collaizzi 1978, and Guba and lincolin (1985) and cannot be replaced |
| **Reviewer #3:** | Adhere to journals guide line on organizing your work | The journal guideline was revised updated through out the text |
|  | Work extensively to be clear grammar and typographical errors throughout the document | Editing was made all over the manuscript |
|  | Title does not specify what kind of danger or suffering nurses are experiencing | Choosing the title encourage readers to read the context to see why the ED is not safe and how it can affect nurse health, hospital, and patient care as well. |
|  | Also the phrase emergency department is not safe anymore it seem like all emergency departments are unsafe which may not be entirely accurate | This title reflects EDS in Jordan. because WPV occurred in high prevalent rates and no serious intervention took in consideration, medical team faced it and some times ended with physical harm that could end with death |
| **Reviewer #3:** | Also on top of your title authors should write the study design | The title in my opinion was written in a way to encourage readers for finding why the ED is not safe |
|  | On part of abstract method the authors need to improve to explain all procedure need | The abstract was updated and some areas were extended and covered . see abstract |
|  | Conclusion the authors should conclude according to result | Conclusion was updated and drown from results. See conclusion section. |
|  | On part of Introduction the authors should revise its very narrow | Introduction was revised , some changes were made  See introduction |
|  | On part of result I comment to include the social demographic table it will be better | The demographic information presented in table 2 |
|  | Also on part of question the authors should improve some of are similar idea and also the quotation the way reported the authors should use the scientific language used in research when reported | Similar ideas and repetition was deleted  See result |
|  | REFFERENCE Should be reversed are not clear in PLOS ONE guideline | The reference list was edited and revised related to PLOS one guidelines |
|  | Red color noted line no 136-138 .also line 144-149 | Were changed , see text please |
|  | Table are not seen | Tables are present at the end of the manuscript |
